# [1,1′-Bis(diphenyl­phosphan­yl)cobalto­cenium]carbonyl­chloridohydrido(triphenyl­phosphane)ruthenium(II) hexa­fluoridophosphate dichloro­methane disolvate

**DOI:** 10.1107/S1600536811044035

**Published:** 2011-11-05

**Authors:** Jian-Guo Hou

**Affiliations:** a Department of Biotic Environment, Nanchang Institute of Technology, Nanchang 330013, People’s Republic of China

## Abstract

In the title compound, [CoRu(C_17_H_14_P)_2_ClH(C_18_H_15_P)(CO)]PF_6_·2CH_2_Cl_2_, the Ru^II^ atom is coordinated by three P atoms from a chelating 1,1′-bis­(diphenyl­phosphan­yl)cobaltocenium ligand and a triphenyl­phosphine ligand, one CO ligand, one Cl atom and one H atom in a distorted octa­hedral geometry. In the cobaltocenium unit, the two cyclo­penta­dienyl rings are almost parallel, making a dihedral angle of 1.2 (3)°. The F atoms of the hexa­fluoridophosphate anion are disordered over two sets of sites, with an occupancy ratio of 0.849 (11):0.151 (11). Intra­molecular C—H⋯Cl hydrogen bonds occur in the complex cation. The complex cations, hexa­fluoridophosphate anions and dichloro­methane solvent mol­ecules are linked by inter­molecular C—H⋯F hydrogen bonds.

## Related literature

For a related structure, see: Santos *et al.* (1994 ▶). 
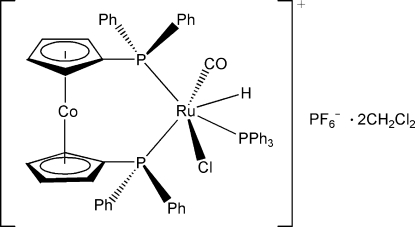


## Experimental

### 

#### Crystal data

[CoRu(C_17_H_14_P)_2_ClH(C_18_H_15_P)(CO)]PF_6_·2CH_2_Cl_2_
*M* *_r_* = 1300.06
Orthorhombic, 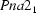

*a* = 22.5676 (19) Å
*b* = 22.0330 (18) Å
*c* = 11.0614 (9) Å
*V* = 5500.1 (8) Å^3^
*Z* = 4
Mo *K*α radiation
μ = 1.00 mm^−1^
*T* = 292 K
0.30 × 0.30 × 0.20 mm

#### Data collection

Bruker APEX CCD diffractometer
Absorption correction: multi-scan (*SADABS*; Sheldrick, 1996 ▶) *T*_min_ = 0.755, *T*_max_ = 0.826
63147 measured reflections
13058 independent reflections
11469 reflections with *I* > 2σ(*I*)
*R*_int_ = 0.065

#### Refinement

*R*[*F* ^2^ > 2σ(*F* ^2^)] = 0.048
*wR*(*F* ^2^) = 0.113
*S* = 1.01
13058 reflections
687 parameters
21 restraints
H atoms treated by a mixture of independent and constrained refinement
Δρ_max_ = 0.77 e Å^−3^
Δρ_min_ = −0.37 e Å^−3^
Absolute structure: Flack (1983 ▶), 6103 Friedel pairs
Flack parameter: −0.002 (16)

### 

Data collection: *SMART* (Bruker, 2007 ▶); cell refinement: *SAINT* (Bruker, 2007 ▶); data reduction: *SAINT*; program(s) used to solve structure: *SHELXS97* (Sheldrick, 2008 ▶); program(s) used to refine structure: *SHELXL97* (Sheldrick, 2008 ▶); molecular graphics: *XP* in *SHELXTL* (Sheldrick, 2008 ▶); software used to prepare material for publication: *SHELXTL*.

## Supplementary Material

Crystal structure: contains datablock(s) I, global. DOI: 10.1107/S1600536811044035/hy2477sup1.cif

Structure factors: contains datablock(s) I. DOI: 10.1107/S1600536811044035/hy2477Isup2.hkl

Additional supplementary materials:  crystallographic information; 3D view; checkCIF report

## Figures and Tables

**Table 1:** Selected bond lengths (Å)

Ru1—P1	2.4827 (10)
Ru1—P2	2.3340 (10)
Ru1—P3	2.3768 (10)
Ru1—Cl1	2.4736 (11)
Ru1—H1*A*	1.80 (3)
Ru1—C35	1.877 (6)

**Table 2:** Hydrogen-bond geometry (Å, °)

*D*—H⋯*A*	*D*—H	H⋯*A*	*D*⋯*A*	*D*—H⋯*A*
C3—H3⋯F2^i^	0.93	2.35	3.230 (9)	157
C7—H7⋯F3^i^	0.93	2.51	3.383 (8)	156
C16—H16⋯Cl1	0.93	2.57	3.414 (5)	151
C37—H37⋯Cl1	0.93	2.77	3.584 (4)	147
C46—H46⋯F2^ii^	0.93	2.54	3.219 (10)	130
C55—H55*A*⋯F3^iii^	0.97	2.42	3.140 (12)	131

Symmetry codes: (i) 
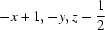
; (ii) 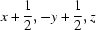; (iii) 
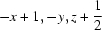
.

**Fig. 1.**
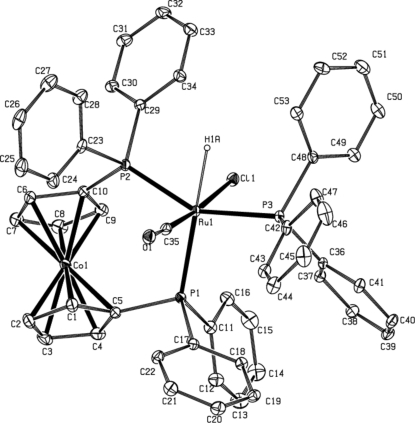
The molecular structure of the title compound with 30% probability displacement ellipsoids.

**Fig. 2.**
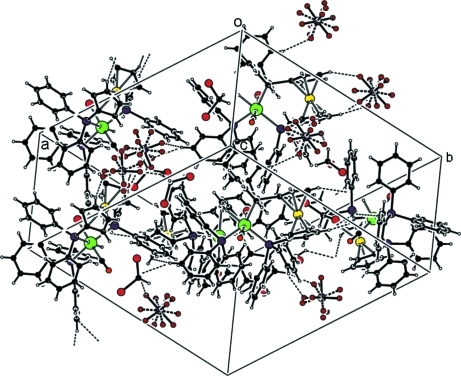
Crystal packing of the title compound. Dashed lines denote hydrogen bonds.

## supplementary crystallographic information

### Comment

The overall geometry of the title compound (Fig. 1) is similar to that of [Ru(CO)HCl(PPh_3_)(dppf)] [dppf = 1,1'-bis(diphenylphosphanyl)ferrocene] (Santos *et al.*, 1994). The distances of Ru—P [2.3340 (10) and 2.4827 (10) Å] (Table 1) are slightly shorter than those found in the dppf analogue [2.392 (4) and 2.518 (4) Å]. The P1—Ru1—P2 chelate angle of 105.47 (3)° in the title compound is larger than that in the dppf analogue [102.2 (1)°]. The cyclopentadienyl (Cp) rings are symmetrically disposed about the Co atom and the dihedral angle between the two Cp rings is 1.2 (3)° [3.1 (5)° in the dppf analogue]. The complex cations, hexafluoridophosphate anions and dichloromethane molecules are linked by intermolecular C—H···F hydrogen bonds (Table 2), as shown in Fig. 2.

### Experimental

A mixture of 1,1'-bis(diphenylphosphanyl)cobaltocenium hexafluoridophosphate (0.562 g, 0.8 mmol) and RuHCl(CO)(PPh_3_)_3_ (0.761 g, 0.8 mmol) was refluxed in CH_2_Cl_2_ (30 ml) for 3 h. The solution was evaporated and the residue was chromatographed on alumina by elution with CH_2_Cl_2_, giving the the title compound as a red solid (yield: 0.724, 82%). Crystals suitable for data collection were obtained by slow evaporation from a dichloromethane and hexane solution at room temperature.

### Refinement

H atoms bound to C atoms were placed in geometrically idealized positions and refined as riding atoms, with C—H = 0.93 (CH) and 0.97 (CH_2_) Å and with *U*_iso_(H) = 1.2*U*_eq_(C). H atom bound to Ru atom was located from a difference Fourier map and refined isotropically. F atoms of the PF_6_ anion are disordered over two sets of positions, with an occupancy ratio of 0.849 (11):0.151 (11). The P—F distances were refined by using *DFIX* command.

### Crystal data

**Table:**

[CoRu(C_17_H_14_P)_2_ClH(C_18_H_15_P)(CO)]PF_6_·2CH_2_Cl_2_	*F*(000) = 2624
*M**_r_* = 1300.06	*D*_x_ = 1.570 Mg m^−^^3^
Orthorhombic, *P**n**a*2_1_	Mo *K*α radiation, λ = 0.71073 Å
Hall symbol: P 2c -2n	Cell parameters from 6748 reflections
*a* = 22.5676 (19) Å	θ = 2.3–26.5°
*b* = 22.0330 (18) Å	µ = 1.00 mm^−^^1^
*c* = 11.0614 (9) Å	*T* = 292 K
*V* = 5500.1 (8) Å^3^	Block, red
*Z* = 4	0.30 × 0.30 × 0.20 mm

### Data collection

**Table:**

Bruker APEX CCD diffractometer	13058 independent reflections
Radiation source: fine-focus sealed tube	11469 reflections with *I* > 2σ(*I*)
graphite	*R*_int_ = 0.065
φ and ω scans	θ_max_ = 28.0°, θ_min_ = 1.3°
Absorption correction: multi-scan (*SADABS*; Sheldrick, 1996)	*h* = −29→29
*T*_min_ = 0.755, *T*_max_ = 0.826	*k* = −28→29
63147 measured reflections	*l* = −14→14

### Refinement

**Table:**

Refinement on *F*^2^	Secondary atom site location: difference Fourier map
Least-squares matrix: full	Hydrogen site location: inferred from neighbouring sites
*R*[*F*^2^ > 2σ(*F*^2^)] = 0.048	H atoms treated by a mixture of independent and constrained refinement
*wR*(*F*^2^) = 0.113	*w* = 1/[σ^2^(*F*_o_^2^) + (0.0706*P*)^2^] where *P* = (*F*_o_^2^ + 2*F*_c_^2^)/3
*S* = 1.01	(Δ/σ)_max_ = 0.003
13058 reflections	Δρ_max_ = 0.77 e Å^−^^3^
687 parameters	Δρ_min_ = −0.37 e Å^−^^3^
21 restraints	Absolute structure: Flack (1983), 6103 Friedel pairs
Primary atom site location: structure-invariant direct methods	Flack parameter: −0.002 (16)

### Fractional atomic coordinates and isotropic or equivalent isotropic displacement parameters (Å^2^)

**Table:**

	*x*	*y*	*z*	*U*_iso_*/*U*_eq_	Occ. (<1)
Ru1	0.807544 (10)	0.117418 (11)	−0.00670 (3)	0.03061 (7)	
Co1	0.64291 (2)	0.01913 (3)	−0.05879 (6)	0.05011 (15)	
O1	0.76093 (14)	0.17429 (16)	0.2091 (3)	0.0562 (8)	
P1	0.78135 (4)	0.01605 (4)	0.07548 (9)	0.0350 (2)	
P2	0.72515 (4)	0.14833 (5)	−0.11840 (9)	0.0354 (2)	
P3	0.90857 (4)	0.12687 (4)	0.05105 (10)	0.03431 (19)	
P4	0.55748 (6)	0.09733 (7)	0.43100 (18)	0.0721 (4)	
Cl1	0.84493 (5)	0.07930 (5)	−0.20272 (10)	0.0481 (2)	
Cl2	0.83826 (13)	0.32376 (11)	0.2555 (3)	0.1414 (10)	
Cl3	0.75697 (11)	0.39945 (9)	0.1218 (2)	0.1104 (6)	
Cl4	0.42940 (16)	0.10480 (11)	1.0041 (3)	0.1572 (11)	
Cl5	0.33733 (17)	0.12977 (12)	0.8247 (3)	0.1432 (10)	
F1	0.5722 (3)	0.1550 (3)	0.5067 (12)	0.183 (5)	0.849 (11)
F2	0.49334 (19)	0.1220 (3)	0.4036 (9)	0.112 (3)	0.849 (11)
F3	0.5436 (3)	0.0395 (2)	0.3517 (6)	0.130 (3)	0.849 (11)
F4	0.5321 (3)	0.0584 (4)	0.5383 (7)	0.143 (3)	0.849 (11)
F5	0.5813 (3)	0.1310 (3)	0.3171 (7)	0.153 (4)	0.849 (11)
F6	0.62033 (18)	0.0705 (2)	0.4637 (9)	0.116 (3)	0.849 (11)
F1'	0.5772 (13)	0.1014 (12)	0.5673 (11)	0.139 (16)*	0.151 (11)
F2'	0.4985 (10)	0.1319 (19)	0.464 (3)	0.109 (16)*	0.151 (11)
F3'	0.530 (2)	0.0986 (16)	0.301 (2)	0.21 (3)*	0.151 (11)
F4'	0.5244 (11)	0.0366 (7)	0.459 (2)	0.085 (9)*	0.151 (11)
F5'	0.5825 (9)	0.1632 (5)	0.410 (2)	0.070 (8)*	0.151 (11)
F6'	0.6186 (7)	0.0706 (10)	0.388 (3)	0.082 (9)*	0.151 (11)
C1	0.6549 (2)	0.0339 (3)	0.1215 (4)	0.0609 (13)	
H1	0.6589	0.0714	0.1593	0.073*	
C2	0.6002 (2)	0.0023 (4)	0.0996 (6)	0.081 (2)	
H2	0.5626	0.0159	0.1207	0.097*	
C3	0.6131 (3)	−0.0525 (3)	0.0413 (6)	0.0809 (19)	
H3	0.5855	−0.0814	0.0172	0.097*	
C4	0.6754 (2)	−0.0567 (2)	0.0250 (5)	0.0625 (14)	
H4	0.6956	−0.0887	−0.0113	0.075*	
C5	0.70195 (17)	−0.0025 (2)	0.0747 (4)	0.0453 (9)	
C6	0.60633 (19)	0.0914 (3)	−0.1470 (5)	0.0566 (12)	
H6	0.5839	0.1224	−0.1131	0.068*	
C7	0.5844 (2)	0.0370 (3)	−0.1941 (5)	0.0705 (16)	
H7	0.5447	0.0257	−0.1954	0.085*	
C8	0.6314 (3)	0.0024 (3)	−0.2388 (5)	0.0682 (15)	
H8	0.6286	−0.0352	−0.2763	0.082*	
C9	0.6848 (2)	0.0357 (2)	−0.2164 (4)	0.0497 (10)	
H9	0.7230	0.0230	−0.2357	0.060*	
C10	0.66962 (18)	0.0909 (2)	−0.1602 (4)	0.0445 (9)	
C11	0.80871 (18)	−0.05791 (19)	0.0207 (4)	0.0428 (10)	
C12	0.8119 (2)	−0.1084 (2)	0.0977 (5)	0.0564 (12)	
H12	0.7989	−0.1052	0.1773	0.068*	
C13	0.8340 (3)	−0.1624 (2)	0.0560 (7)	0.0755 (16)	
H13	0.8371	−0.1953	0.1085	0.091*	
C14	0.8517 (3)	−0.1687 (3)	−0.0608 (7)	0.0799 (17)	
H14	0.8666	−0.2056	−0.0880	0.096*	
C15	0.8472 (3)	−0.1195 (3)	−0.1397 (6)	0.0765 (18)	
H15	0.8584	−0.1236	−0.2202	0.092*	
C16	0.8262 (2)	−0.0651 (2)	−0.0975 (5)	0.0541 (11)	
H16	0.8237	−0.0323	−0.1501	0.065*	
C17	0.79845 (17)	0.01169 (17)	0.2384 (4)	0.0377 (8)	
C18	0.85440 (18)	−0.00775 (18)	0.2743 (4)	0.0427 (9)	
H18	0.8813	−0.0211	0.2165	0.051*	
C19	0.8705 (2)	−0.0074 (2)	0.3957 (4)	0.0511 (10)	
H19	0.9077	−0.0213	0.4187	0.061*	
C20	0.8317 (2)	0.0133 (2)	0.4814 (4)	0.0557 (11)	
H20	0.8425	0.0135	0.5626	0.067*	
C21	0.7770 (2)	0.0337 (2)	0.4468 (5)	0.0560 (11)	
H21	0.7510	0.0487	0.5049	0.067*	
C22	0.7597 (2)	0.0325 (2)	0.3266 (4)	0.0488 (10)	
H22	0.7220	0.0457	0.3049	0.059*	
C23	0.68043 (17)	0.2102 (2)	−0.0536 (4)	0.0445 (9)	
C24	0.6469 (2)	0.2018 (3)	0.0477 (5)	0.0652 (13)	
H24	0.6458	0.1635	0.0831	0.078*	
C25	0.6146 (2)	0.2482 (3)	0.0995 (6)	0.0781 (17)	
H25	0.5914	0.2409	0.1675	0.094*	
C26	0.6167 (3)	0.3041 (3)	0.0512 (6)	0.0813 (19)	
H26	0.5949	0.3354	0.0857	0.098*	
C27	0.6514 (3)	0.3153 (3)	−0.0504 (6)	0.0809 (18)	
H27	0.6534	0.3541	−0.0830	0.097*	
C28	0.6831 (2)	0.2677 (2)	−0.1028 (5)	0.0634 (13)	
H28	0.7061	0.2748	−0.1712	0.076*	
C29	0.74319 (17)	0.17725 (18)	−0.2695 (4)	0.0382 (8)	
C30	0.7073 (2)	0.16652 (19)	−0.3673 (4)	0.0471 (10)	
H30	0.6726	0.1443	−0.3576	0.056*	
C31	0.7225 (2)	0.1889 (2)	−0.4814 (4)	0.0599 (12)	
H31	0.6990	0.1797	−0.5480	0.072*	
C32	0.7717 (2)	0.2239 (2)	−0.4953 (5)	0.0605 (12)	
H32	0.7813	0.2393	−0.5711	0.073*	
C33	0.8066 (2)	0.2365 (2)	−0.3991 (5)	0.0600 (13)	
H33	0.8397	0.2611	−0.4089	0.072*	
C34	0.7934 (2)	0.2129 (2)	−0.2860 (4)	0.0502 (10)	
H34	0.8183	0.2210	−0.2208	0.060*	
C35	0.77785 (19)	0.1531 (2)	0.1350 (5)	0.0460 (10)	
C36	0.95425 (15)	0.05945 (17)	0.0759 (4)	0.0364 (8)	
C37	0.94499 (17)	0.00857 (17)	0.0058 (4)	0.0415 (8)	
H37	0.9156	0.0091	−0.0531	0.050*	
C38	0.9788 (2)	−0.04324 (19)	0.0216 (4)	0.0510 (11)	
H38	0.9722	−0.0771	−0.0266	0.061*	
C39	1.0221 (2)	−0.0445 (2)	0.1085 (4)	0.0552 (12)	
H39	1.0442	−0.0796	0.1203	0.066*	
C40	1.0328 (2)	0.0054 (2)	0.1776 (4)	0.0539 (11)	
H40	1.0627	0.0046	0.2355	0.065*	
C41	0.99896 (18)	0.0579 (2)	0.1619 (4)	0.0473 (10)	
H41	1.0064	0.0919	0.2092	0.057*	
C42	0.92323 (16)	0.16867 (19)	0.1895 (4)	0.0439 (9)	
C43	0.8969 (2)	0.1482 (2)	0.2964 (4)	0.0474 (10)	
H43	0.8687	0.1175	0.2928	0.057*	
C44	0.9117 (2)	0.1726 (3)	0.4066 (5)	0.0637 (13)	
H44	0.8944	0.1578	0.4771	0.076*	
C45	0.9517 (3)	0.2187 (3)	0.4118 (6)	0.0833 (19)	
H45	0.9616	0.2354	0.4863	0.100*	
C46	0.9776 (3)	0.2407 (3)	0.3096 (7)	0.093 (2)	
H46	1.0049	0.2722	0.3154	0.112*	
C47	0.9640 (2)	0.2167 (3)	0.1971 (6)	0.0685 (15)	
H47	0.9816	0.2322	0.1276	0.082*	
C48	0.95041 (16)	0.16744 (18)	−0.0672 (4)	0.0421 (8)	
C49	0.9954 (2)	0.1391 (2)	−0.1317 (4)	0.0539 (11)	
H49	1.0055	0.0991	−0.1138	0.065*	
C50	1.0254 (2)	0.1694 (3)	−0.2218 (5)	0.0656 (14)	
H50	1.0549	0.1496	−0.2651	0.079*	
C51	1.0120 (3)	0.2281 (3)	−0.2481 (6)	0.0745 (15)	
H51	1.0328	0.2486	−0.3081	0.089*	
C52	0.9678 (3)	0.2571 (3)	−0.1859 (7)	0.0850 (19)	
H52	0.9585	0.2972	−0.2039	0.102*	
C53	0.9365 (2)	0.2265 (2)	−0.0952 (6)	0.0655 (14)	
H53	0.9062	0.2462	−0.0539	0.079*	
C54	0.7753 (4)	0.3264 (3)	0.1644 (11)	0.122 (3)	
H54A	0.7820	0.3022	0.0925	0.147*	
H54B	0.7422	0.3087	0.2079	0.147*	
C55	0.3782 (6)	0.0762 (4)	0.8963 (12)	0.170 (5)	
H55A	0.3998	0.0532	0.8361	0.204*	
H55B	0.3514	0.0484	0.9369	0.204*	
H1A	0.827 (2)	0.1904 (12)	−0.069 (5)	0.074 (15)*	

### Atomic displacement parameters (Å^2^)

**Table:**

	*U*^11^	*U*^22^	*U*^33^	*U*^12^	*U*^13^	*U*^23^
Ru1	0.02449 (11)	0.03693 (13)	0.03043 (13)	0.00218 (10)	0.00054 (12)	−0.00293 (13)
Co1	0.0344 (3)	0.0712 (4)	0.0447 (3)	−0.0147 (3)	−0.0085 (2)	0.0130 (3)
O1	0.0506 (18)	0.065 (2)	0.053 (2)	0.0084 (15)	0.0015 (16)	−0.0015 (17)
P1	0.0318 (4)	0.0421 (5)	0.0311 (5)	−0.0036 (4)	−0.0027 (4)	0.0000 (4)
P2	0.0280 (4)	0.0456 (5)	0.0326 (5)	0.0035 (4)	0.0006 (4)	0.0009 (4)
P3	0.0261 (4)	0.0372 (5)	0.0397 (5)	0.0002 (3)	−0.0014 (4)	−0.0030 (4)
P4	0.0455 (7)	0.0763 (9)	0.0946 (11)	−0.0020 (7)	−0.0003 (7)	0.0056 (9)
Cl1	0.0452 (5)	0.0550 (6)	0.0441 (5)	0.0093 (4)	0.0032 (4)	−0.0040 (4)
Cl2	0.1271 (18)	0.1176 (17)	0.179 (3)	0.0031 (14)	0.0063 (18)	0.0646 (18)
Cl3	0.1264 (16)	0.0810 (10)	0.1236 (17)	0.0072 (11)	0.0042 (13)	0.0066 (11)
Cl4	0.222 (3)	0.1184 (16)	0.131 (2)	0.0118 (18)	0.028 (3)	0.0147 (18)
Cl5	0.187 (3)	0.1144 (17)	0.128 (2)	−0.0131 (18)	0.011 (2)	−0.0096 (15)
F1	0.106 (4)	0.158 (6)	0.286 (14)	0.021 (4)	−0.034 (6)	−0.139 (8)
F2	0.053 (2)	0.128 (5)	0.157 (7)	0.006 (2)	0.007 (3)	0.065 (5)
F3	0.140 (5)	0.108 (4)	0.141 (6)	−0.024 (3)	−0.041 (4)	−0.013 (4)
F4	0.098 (4)	0.214 (7)	0.116 (5)	0.014 (4)	0.009 (3)	0.073 (5)
F5	0.134 (5)	0.144 (5)	0.181 (8)	0.007 (4)	0.072 (5)	0.065 (5)
F6	0.054 (2)	0.106 (3)	0.189 (8)	0.010 (2)	−0.026 (3)	−0.015 (4)
C1	0.040 (2)	0.106 (4)	0.037 (2)	−0.001 (2)	−0.0023 (18)	0.013 (2)
C2	0.039 (3)	0.143 (6)	0.061 (4)	−0.013 (3)	0.000 (2)	0.042 (4)
C3	0.062 (3)	0.099 (4)	0.081 (4)	−0.042 (3)	−0.021 (3)	0.037 (4)
C4	0.061 (3)	0.065 (3)	0.062 (3)	−0.026 (2)	−0.024 (2)	0.020 (2)
C5	0.0358 (19)	0.062 (3)	0.038 (2)	−0.0064 (18)	−0.0068 (17)	0.0129 (19)
C6	0.037 (2)	0.079 (3)	0.053 (3)	−0.002 (2)	−0.0076 (19)	0.018 (2)
C7	0.049 (3)	0.099 (4)	0.063 (3)	−0.028 (3)	−0.031 (3)	0.025 (3)
C8	0.077 (4)	0.080 (3)	0.047 (3)	−0.025 (3)	−0.021 (3)	0.004 (3)
C9	0.051 (2)	0.057 (3)	0.041 (2)	−0.010 (2)	−0.0069 (19)	0.0016 (19)
C10	0.0357 (19)	0.061 (3)	0.037 (2)	−0.0056 (19)	−0.0066 (16)	0.0105 (18)
C11	0.046 (2)	0.047 (2)	0.036 (3)	−0.0072 (17)	−0.0067 (16)	−0.0079 (15)
C12	0.068 (3)	0.047 (2)	0.053 (3)	−0.010 (2)	−0.006 (2)	−0.004 (2)
C13	0.096 (4)	0.043 (3)	0.088 (4)	−0.008 (3)	−0.003 (4)	−0.008 (3)
C14	0.094 (4)	0.050 (3)	0.096 (5)	0.003 (3)	0.002 (4)	−0.023 (3)
C15	0.088 (4)	0.069 (4)	0.073 (4)	−0.010 (3)	0.012 (3)	−0.032 (3)
C16	0.057 (3)	0.051 (3)	0.055 (3)	−0.011 (2)	−0.001 (2)	−0.006 (2)
C17	0.038 (2)	0.0389 (19)	0.036 (2)	−0.0054 (15)	−0.0045 (15)	0.0009 (15)
C18	0.043 (2)	0.046 (2)	0.039 (2)	−0.0031 (16)	−0.0024 (16)	−0.0051 (17)
C19	0.055 (3)	0.049 (2)	0.049 (3)	−0.001 (2)	−0.015 (2)	0.0035 (19)
C20	0.066 (3)	0.065 (3)	0.036 (2)	−0.003 (2)	−0.009 (2)	−0.003 (2)
C21	0.062 (3)	0.064 (3)	0.042 (2)	0.006 (2)	0.009 (2)	−0.010 (2)
C22	0.046 (2)	0.058 (3)	0.042 (2)	0.0084 (19)	−0.0040 (18)	−0.0006 (19)
C23	0.0315 (18)	0.060 (3)	0.042 (2)	0.0141 (17)	−0.0060 (16)	−0.0057 (19)
C24	0.049 (3)	0.085 (3)	0.062 (3)	0.013 (2)	0.015 (2)	0.001 (3)
C25	0.055 (3)	0.117 (5)	0.062 (3)	0.026 (3)	0.005 (3)	−0.012 (3)
C26	0.065 (3)	0.103 (5)	0.076 (4)	0.046 (3)	−0.011 (3)	−0.036 (4)
C27	0.094 (4)	0.069 (3)	0.080 (4)	0.039 (3)	−0.007 (4)	−0.014 (3)
C28	0.069 (3)	0.066 (3)	0.055 (3)	0.022 (3)	−0.001 (2)	−0.008 (2)
C29	0.040 (2)	0.040 (2)	0.035 (2)	0.0078 (16)	0.0061 (16)	0.0026 (15)
C30	0.054 (2)	0.047 (2)	0.040 (2)	0.0026 (19)	−0.0006 (19)	0.0013 (18)
C31	0.078 (3)	0.062 (3)	0.039 (3)	0.011 (2)	−0.002 (2)	0.003 (2)
C32	0.075 (3)	0.065 (3)	0.041 (3)	0.009 (2)	0.018 (3)	0.012 (2)
C33	0.059 (3)	0.061 (3)	0.060 (3)	−0.003 (2)	0.012 (2)	0.007 (2)
C34	0.046 (2)	0.062 (3)	0.043 (2)	−0.003 (2)	0.0042 (18)	0.007 (2)
C35	0.039 (2)	0.046 (2)	0.053 (3)	−0.0035 (18)	−0.013 (2)	0.007 (2)
C36	0.0266 (16)	0.0425 (19)	0.040 (2)	0.0051 (14)	0.0034 (15)	0.0043 (16)
C37	0.0382 (18)	0.0457 (19)	0.041 (2)	0.0037 (15)	−0.0043 (18)	−0.0010 (19)
C38	0.053 (2)	0.041 (2)	0.060 (3)	0.0029 (18)	0.0057 (19)	−0.0033 (18)
C39	0.054 (3)	0.057 (3)	0.056 (3)	0.023 (2)	0.008 (2)	0.013 (2)
C40	0.045 (2)	0.072 (3)	0.045 (2)	0.019 (2)	−0.0067 (19)	0.005 (2)
C41	0.038 (2)	0.058 (2)	0.045 (2)	0.0104 (18)	−0.0068 (17)	−0.0073 (19)
C42	0.0304 (18)	0.048 (2)	0.053 (2)	0.0021 (16)	−0.0068 (17)	−0.0169 (19)
C43	0.048 (2)	0.048 (2)	0.047 (2)	0.0059 (18)	−0.0045 (19)	−0.0071 (19)
C44	0.062 (3)	0.078 (3)	0.051 (3)	0.015 (3)	−0.009 (2)	−0.021 (2)
C45	0.068 (3)	0.111 (5)	0.070 (4)	−0.004 (3)	−0.005 (3)	−0.044 (4)
C46	0.066 (4)	0.108 (5)	0.105 (5)	−0.032 (3)	0.002 (4)	−0.064 (4)
C47	0.052 (3)	0.070 (3)	0.084 (4)	−0.022 (2)	0.015 (3)	−0.036 (3)
C48	0.0305 (17)	0.048 (2)	0.048 (2)	−0.0059 (16)	−0.0017 (17)	−0.0001 (19)
C49	0.051 (2)	0.058 (3)	0.054 (3)	−0.005 (2)	0.007 (2)	−0.001 (2)
C50	0.057 (3)	0.075 (3)	0.065 (3)	−0.014 (2)	0.019 (2)	−0.006 (3)
C51	0.068 (3)	0.091 (4)	0.064 (3)	−0.024 (3)	0.008 (3)	0.014 (3)
C52	0.075 (4)	0.060 (3)	0.120 (5)	−0.010 (3)	0.016 (4)	0.039 (4)
C53	0.048 (3)	0.060 (3)	0.088 (4)	0.001 (2)	0.012 (2)	0.017 (3)
C54	0.127 (7)	0.070 (4)	0.170 (10)	0.001 (4)	−0.015 (7)	0.003 (5)
C55	0.255 (14)	0.084 (6)	0.171 (12)	0.009 (7)	0.000 (11)	−0.053 (7)

### Geometric parameters (Å, °)

**Table:**

Ru1—P1	2.4827 (10)	C17—C22	1.389 (6)
Ru1—P2	2.3340 (10)	C17—C18	1.391 (6)
Ru1—P3	2.3768 (10)	C18—C19	1.391 (6)
Ru1—Cl1	2.4736 (11)	C18—H18	0.9300
Ru1—H1A	1.80 (3)	C19—C20	1.369 (7)
Ru1—C35	1.877 (6)	C19—H19	0.9300
Co1—C9	2.017 (5)	C20—C21	1.370 (7)
Co1—C10	2.030 (4)	C20—H20	0.9300
Co1—C2	2.034 (5)	C21—C22	1.387 (7)
Co1—C7	2.034 (5)	C21—H21	0.9300
Co1—C1	2.039 (5)	C22—H22	0.9300
Co1—C3	2.041 (5)	C23—C24	1.365 (7)
Co1—C6	2.041 (5)	C23—C28	1.381 (7)
Co1—C8	2.042 (5)	C24—C25	1.382 (8)
Co1—C5	2.045 (4)	C24—H24	0.9300
Co1—C4	2.046 (5)	C25—C26	1.342 (9)
O1—C35	1.017 (6)	C25—H25	0.9300
P1—C5	1.838 (4)	C26—C27	1.391 (10)
P1—C11	1.845 (4)	C26—H26	0.9300
P1—C17	1.845 (4)	C27—C28	1.396 (7)
P2—C29	1.835 (4)	C27—H27	0.9300
P2—C10	1.841 (4)	C28—H28	0.9300
P2—C23	1.841 (4)	C29—C30	1.372 (6)
P3—C42	1.817 (4)	C29—C34	1.392 (6)
P3—C36	1.829 (4)	C30—C31	1.398 (6)
P3—C48	1.845 (4)	C30—H30	0.9300
P4—F5	1.557 (5)	C31—C32	1.360 (7)
P4—F1	1.557 (5)	C31—H31	0.9300
P4—F4'	1.563 (8)	C32—C33	1.353 (8)
P4—F3'	1.568 (8)	C32—H32	0.9300
P4—F4	1.572 (5)	C33—C34	1.386 (7)
P4—F6'	1.573 (9)	C33—H33	0.9300
P4—F5'	1.574 (8)	C34—H34	0.9300
P4—F1'	1.574 (8)	C36—C37	1.379 (6)
P4—F2	1.576 (4)	C36—C41	1.387 (5)
P4—F2'	1.576 (9)	C37—C38	1.385 (6)
P4—F3	1.578 (5)	C37—H37	0.9300
P4—F6	1.579 (4)	C38—C39	1.370 (7)
Cl2—C54	1.744 (10)	C38—H38	0.9300
Cl3—C54	1.727 (8)	C39—C40	1.362 (7)
Cl4—C55	1.776 (12)	C39—H39	0.9300
Cl5—C55	1.694 (12)	C40—C41	1.396 (6)
C1—C5	1.429 (7)	C40—H40	0.9300
C1—C2	1.438 (8)	C41—H41	0.9300
C1—H1	0.9300	C42—C43	1.398 (7)
C2—C3	1.399 (10)	C42—C47	1.404 (6)
C2—H2	0.9300	C43—C44	1.374 (7)
C3—C4	1.420 (9)	C43—H43	0.9300
C3—H3	0.9300	C44—C45	1.361 (8)
C4—C5	1.444 (7)	C44—H44	0.9300
C4—H4	0.9300	C45—C46	1.361 (10)
C6—C7	1.396 (8)	C45—H45	0.9300
C6—C10	1.436 (6)	C46—C47	1.387 (8)
C6—H6	0.9300	C46—H46	0.9300
C7—C8	1.398 (9)	C47—H47	0.9300
C7—H7	0.9300	C48—C53	1.373 (6)
C8—C9	1.432 (7)	C48—C49	1.390 (6)
C8—H8	0.9300	C49—C50	1.377 (7)
C9—C10	1.409 (7)	C49—H49	0.9300
C9—H9	0.9300	C50—C51	1.361 (8)
C11—C16	1.375 (7)	C50—H50	0.9300
C11—C12	1.402 (7)	C51—C52	1.369 (9)
C12—C13	1.372 (7)	C51—H51	0.9300
C12—H12	0.9300	C52—C53	1.400 (8)
C13—C14	1.359 (10)	C52—H52	0.9300
C13—H13	0.9300	C53—H53	0.9300
C14—C15	1.396 (10)	C54—H54A	0.9700
C14—H14	0.9300	C54—H54B	0.9700
C15—C16	1.370 (7)	C55—H55A	0.9700
C15—H15	0.9300	C55—H55B	0.9700
C16—H16	0.9300		
			
C35—Ru1—P2	92.03 (12)	C9—C8—H8	126.3
C35—Ru1—P3	94.66 (12)	Co1—C8—H8	127.2
P2—Ru1—P3	151.74 (4)	C10—C9—C8	108.2 (5)
C35—Ru1—Cl1	174.78 (13)	C10—C9—Co1	70.1 (3)
P2—Ru1—Cl1	84.65 (4)	C8—C9—Co1	70.3 (3)
P3—Ru1—Cl1	86.45 (4)	C10—C9—H9	125.9
C35—Ru1—P1	89.21 (13)	C8—C9—H9	125.9
P2—Ru1—P1	105.47 (3)	Co1—C9—H9	125.3
P3—Ru1—P1	102.05 (3)	C9—C10—C6	107.1 (4)
Cl1—Ru1—P1	95.55 (4)	C9—C10—P2	122.6 (3)
C35—Ru1—H1A	91.6 (19)	C6—C10—P2	130.3 (4)
P2—Ru1—H1A	74.2 (17)	C9—C10—Co1	69.2 (3)
P3—Ru1—H1A	78.2 (17)	C6—C10—Co1	69.8 (3)
Cl1—Ru1—H1A	84 (2)	P2—C10—Co1	126.8 (2)
P1—Ru1—H1A	179 (2)	C16—C11—C12	118.1 (4)
C9—Co1—C10	40.74 (19)	C16—C11—P1	120.7 (4)
C9—Co1—C2	179.7 (2)	C12—C11—P1	121.2 (3)
C10—Co1—C2	139.4 (3)	C13—C12—C11	120.2 (5)
C9—Co1—C7	68.5 (2)	C13—C12—H12	119.9
C10—Co1—C7	68.61 (18)	C11—C12—H12	119.9
C2—Co1—C7	111.2 (2)	C14—C13—C12	121.0 (6)
C9—Co1—C1	139.00 (19)	C14—C13—H13	119.5
C10—Co1—C1	112.1 (2)	C12—C13—H13	119.5
C2—Co1—C1	41.3 (2)	C13—C14—C15	119.5 (6)
C7—Co1—C1	140.7 (2)	C13—C14—H14	120.2
C9—Co1—C3	139.7 (3)	C15—C14—H14	120.2
C10—Co1—C3	178.0 (2)	C16—C15—C14	119.5 (6)
C2—Co1—C3	40.1 (3)	C16—C15—H15	120.3
C7—Co1—C3	109.6 (2)	C14—C15—H15	120.3
C1—Co1—C3	68.7 (3)	C15—C16—C11	121.6 (5)
C9—Co1—C6	68.6 (2)	C15—C16—H16	119.2
C10—Co1—C6	41.30 (18)	C11—C16—H16	119.2
C2—Co1—C6	111.3 (3)	C22—C17—C18	118.2 (4)
C7—Co1—C6	40.1 (2)	C22—C17—P1	122.5 (3)
C1—Co1—C6	113.3 (2)	C18—C17—P1	119.0 (3)
C3—Co1—C6	136.8 (2)	C19—C18—C17	120.8 (4)
C9—Co1—C8	41.3 (2)	C19—C18—H18	119.6
C10—Co1—C8	68.8 (2)	C17—C18—H18	119.6
C2—Co1—C8	138.3 (3)	C20—C19—C18	120.2 (4)
C7—Co1—C8	40.1 (3)	C20—C19—H19	119.9
C1—Co1—C8	178.8 (3)	C18—C19—H19	119.9
C3—Co1—C8	110.4 (3)	C19—C20—C21	119.5 (4)
C6—Co1—C8	67.9 (2)	C19—C20—H20	120.2
C9—Co1—C5	111.13 (18)	C21—C20—H20	120.2
C10—Co1—C5	112.78 (16)	C20—C21—C22	121.0 (4)
C2—Co1—C5	69.2 (2)	C20—C21—H21	119.5
C7—Co1—C5	177.6 (2)	C22—C21—H21	119.5
C1—Co1—C5	40.97 (19)	C21—C22—C17	120.2 (4)
C3—Co1—C5	69.06 (19)	C21—C22—H22	119.9
C6—Co1—C5	142.2 (2)	C17—C22—H22	119.9
C8—Co1—C5	138.1 (2)	C24—C23—C28	118.2 (4)
C9—Co1—C4	111.7 (2)	C24—C23—P2	121.6 (4)
C10—Co1—C4	141.21 (19)	C28—C23—P2	120.1 (4)
C2—Co1—C4	68.3 (3)	C23—C24—C25	122.2 (6)
C7—Co1—C4	136.4 (2)	C23—C24—H24	118.9
C1—Co1—C4	68.9 (2)	C25—C24—H24	118.9
C3—Co1—C4	40.7 (2)	C26—C25—C24	119.7 (6)
C6—Co1—C4	176.34 (19)	C26—C25—H25	120.2
C8—Co1—C4	109.9 (2)	C24—C25—H25	120.2
C5—Co1—C4	41.34 (18)	C25—C26—C27	120.3 (5)
C5—P1—C11	97.4 (2)	C25—C26—H26	119.9
C5—P1—C17	101.36 (19)	C27—C26—H26	119.9
C11—P1—C17	101.81 (18)	C26—C27—C28	119.4 (6)
C5—P1—Ru1	115.51 (14)	C26—C27—H27	120.3
C11—P1—Ru1	126.49 (14)	C28—C27—H27	120.3
C17—P1—Ru1	110.77 (13)	C23—C28—C27	120.2 (6)
C29—P2—C10	99.27 (18)	C23—C28—H28	119.9
C29—P2—C23	102.7 (2)	C27—C28—H28	119.9
C10—P2—C23	103.5 (2)	C30—C29—C34	118.4 (4)
C29—P2—Ru1	114.02 (13)	C30—C29—P2	121.8 (3)
C10—P2—Ru1	118.34 (14)	C34—C29—P2	119.7 (3)
C23—P2—Ru1	116.53 (14)	C29—C30—C31	120.4 (4)
C42—P3—C36	100.51 (19)	C29—C30—H30	119.8
C42—P3—C48	105.0 (2)	C31—C30—H30	119.8
C36—P3—C48	102.22 (17)	C32—C31—C30	120.2 (5)
C42—P3—Ru1	116.45 (13)	C32—C31—H31	119.9
C36—P3—Ru1	120.66 (13)	C30—C31—H31	119.9
C48—P3—Ru1	110.05 (13)	C33—C32—C31	120.2 (5)
F5—P4—F1	88.4 (5)	C33—C32—H32	119.9
F4'—P4—F3'	90.2 (5)	C31—C32—H32	119.9
F5—P4—F4	174.9 (5)	C32—C33—C34	120.6 (5)
F1—P4—F4	96.7 (6)	C32—C33—H33	119.7
F4'—P4—F6'	99.1 (14)	C34—C33—H33	119.7
F3'—P4—F6'	95 (3)	C33—C34—C29	120.3 (5)
F4'—P4—F5'	171.6 (13)	C33—C34—H34	119.9
F3'—P4—F5'	89.7 (5)	C29—C34—H34	119.9
F6'—P4—F5'	89.3 (6)	O1—C35—Ru1	177.0 (4)
F4'—P4—F1'	89.7 (5)	C37—C36—C41	118.4 (4)
F3'—P4—F1'	172 (3)	C37—C36—P3	119.4 (3)
F6'—P4—F1'	93.6 (16)	C41—C36—P3	122.2 (3)
F5'—P4—F1'	89.2 (5)	C36—C37—C38	121.0 (4)
F5—P4—F2	89.8 (4)	C36—C37—H37	119.5
F1—P4—F2	91.0 (5)	C38—C37—H37	119.5
F4—P4—F2	89.9 (3)	C39—C38—C37	119.9 (4)
F4'—P4—F2'	88 (2)	C39—C38—H38	120.1
F3'—P4—F2'	82 (3)	C37—C38—H38	120.1
F6'—P4—F2'	172.4 (19)	C40—C39—C38	120.3 (4)
F5'—P4—F2'	83.8 (19)	C40—C39—H39	119.9
F1'—P4—F2'	89.3 (6)	C38—C39—H39	119.9
F5—P4—F3	90.2 (4)	C39—C40—C41	120.1 (4)
F1—P4—F3	178.6 (5)	C39—C40—H40	119.9
F4—P4—F3	84.7 (4)	C41—C40—H40	119.9
F2—P4—F3	89.4 (5)	C36—C41—C40	120.2 (4)
F5—P4—F6	93.1 (4)	C36—C41—H41	119.9
F1—P4—F6	89.5 (3)	C40—C41—H41	119.9
F4—P4—F6	87.1 (4)	C43—C42—C47	118.1 (4)
F2—P4—F6	177.0 (4)	C43—C42—P3	118.2 (3)
F3—P4—F6	90.2 (3)	C47—C42—P3	123.5 (4)
C5—C1—C2	107.7 (6)	C44—C43—C42	121.4 (4)
C5—C1—Co1	69.8 (3)	C44—C43—H43	119.3
C2—C1—Co1	69.2 (3)	C42—C43—H43	119.3
C5—C1—H1	126.1	C45—C44—C43	119.4 (6)
C2—C1—H1	126.1	C45—C44—H44	120.3
Co1—C1—H1	126.5	C43—C44—H44	120.3
C3—C2—C1	108.5 (5)	C44—C45—C46	121.1 (5)
C3—C2—Co1	70.2 (4)	C44—C45—H45	119.5
C1—C2—Co1	69.5 (3)	C46—C45—H45	119.5
C3—C2—H2	125.8	C45—C46—C47	121.0 (5)
C1—C2—H2	125.8	C45—C46—H46	119.5
Co1—C2—H2	126.1	C47—C46—H46	119.5
C2—C3—C4	108.8 (5)	C46—C47—C42	119.1 (6)
C2—C3—Co1	69.7 (3)	C46—C47—H47	120.5
C4—C3—Co1	69.9 (3)	C42—C47—H47	120.5
C2—C3—H3	125.6	C53—C48—C49	118.5 (4)
C4—C3—H3	125.6	C53—C48—P3	120.1 (4)
Co1—C3—H3	126.4	C49—C48—P3	121.3 (3)
C3—C4—C5	107.9 (5)	C50—C49—C48	120.8 (5)
C3—C4—Co1	69.5 (3)	C50—C49—H49	119.6
C5—C4—Co1	69.3 (3)	C48—C49—H49	119.6
C3—C4—H4	126.0	C51—C50—C49	120.5 (5)
C5—C4—H4	126.0	C51—C50—H50	119.8
Co1—C4—H4	126.8	C49—C50—H50	119.8
C1—C5—C4	107.1 (4)	C50—C51—C52	119.8 (5)
C1—C5—P1	126.7 (4)	C50—C51—H51	120.1
C4—C5—P1	126.2 (4)	C52—C51—H51	120.1
C1—C5—Co1	69.3 (2)	C51—C52—C53	120.2 (5)
C4—C5—Co1	69.4 (3)	C51—C52—H52	119.9
P1—C5—Co1	125.9 (2)	C53—C52—H52	119.9
C7—C6—C10	107.9 (5)	C48—C53—C52	120.2 (5)
C7—C6—Co1	69.7 (3)	C48—C53—H53	119.9
C10—C6—Co1	68.9 (3)	C52—C53—H53	119.9
C7—C6—H6	126.0	Cl3—C54—Cl2	112.6 (5)
C10—C6—H6	126.0	Cl3—C54—H54A	109.1
Co1—C6—H6	126.9	Cl2—C54—H54A	109.1
C6—C7—C8	109.3 (4)	Cl3—C54—H54B	109.1
C6—C7—Co1	70.2 (3)	Cl2—C54—H54B	109.1
C8—C7—Co1	70.2 (3)	H54A—C54—H54B	107.8
C6—C7—H7	125.4	Cl5—C55—Cl4	114.9 (5)
C8—C7—H7	125.4	Cl5—C55—H55A	108.5
Co1—C7—H7	125.8	Cl4—C55—H55A	108.5
C7—C8—C9	107.4 (5)	Cl5—C55—H55B	108.5
C7—C8—Co1	69.7 (3)	Cl4—C55—H55B	108.5
C9—C8—Co1	68.4 (3)	H55A—C55—H55B	107.5
C7—C8—H8	126.3		
			
C35—Ru1—P1—C5	74.3 (2)	C6—C7—C8—C9	1.4 (6)
P2—Ru1—P1—C5	−17.62 (17)	Co1—C7—C8—C9	−58.2 (3)
P3—Ru1—P1—C5	168.87 (17)	C6—C7—C8—Co1	59.6 (4)
Cl1—Ru1—P1—C5	−103.59 (17)	C9—Co1—C8—C7	−119.3 (5)
C35—Ru1—P1—C11	−163.8 (2)	C10—Co1—C8—C7	−81.5 (3)
P2—Ru1—P1—C11	104.35 (16)	C2—Co1—C8—C7	60.6 (5)
P3—Ru1—P1—C11	−69.17 (16)	C3—Co1—C8—C7	96.6 (4)
Cl1—Ru1—P1—C11	18.38 (16)	C6—Co1—C8—C7	−36.9 (3)
C35—Ru1—P1—C17	−40.22 (18)	C5—Co1—C8—C7	177.5 (3)
P2—Ru1—P1—C17	−132.10 (14)	C4—Co1—C8—C7	140.0 (3)
P3—Ru1—P1—C17	54.38 (14)	C10—Co1—C8—C9	37.8 (3)
Cl1—Ru1—P1—C17	141.93 (14)	C2—Co1—C8—C9	179.9 (4)
C35—Ru1—P2—C29	138.15 (18)	C7—Co1—C8—C9	119.3 (5)
P3—Ru1—P2—C29	34.40 (17)	C3—Co1—C8—C9	−144.1 (3)
Cl1—Ru1—P2—C29	−37.81 (14)	C6—Co1—C8—C9	82.4 (3)
P1—Ru1—P2—C29	−132.11 (14)	C5—Co1—C8—C9	−63.2 (4)
C35—Ru1—P2—C10	−105.7 (2)	C4—Co1—C8—C9	−100.7 (3)
P3—Ru1—P2—C10	150.54 (17)	C7—C8—C9—C10	−1.1 (6)
Cl1—Ru1—P2—C10	78.33 (17)	Co1—C8—C9—C10	−60.1 (3)
P1—Ru1—P2—C10	−15.96 (17)	C7—C8—C9—Co1	59.0 (4)
C35—Ru1—P2—C23	18.8 (2)	C7—Co1—C9—C10	81.7 (3)
P3—Ru1—P2—C23	−84.99 (19)	C1—Co1—C9—C10	−62.9 (4)
Cl1—Ru1—P2—C23	−157.20 (17)	C3—Co1—C9—C10	177.1 (3)
P1—Ru1—P2—C23	108.50 (17)	C6—Co1—C9—C10	38.6 (3)
C35—Ru1—P3—C42	−6.2 (2)	C8—Co1—C9—C10	118.9 (4)
P2—Ru1—P3—C42	96.89 (18)	C5—Co1—C9—C10	−100.8 (3)
Cl1—Ru1—P3—C42	168.67 (17)	C4—Co1—C9—C10	−145.4 (2)
P1—Ru1—P3—C42	−96.41 (17)	C10—Co1—C9—C8	−118.9 (4)
C35—Ru1—P3—C36	115.9 (2)	C7—Co1—C9—C8	−37.1 (4)
P2—Ru1—P3—C36	−140.99 (15)	C1—Co1—C9—C8	178.2 (4)
Cl1—Ru1—P3—C36	−69.21 (15)	C3—Co1—C9—C8	58.2 (5)
P1—Ru1—P3—C36	25.72 (15)	C6—Co1—C9—C8	−80.3 (4)
C35—Ru1—P3—C48	−125.53 (19)	C5—Co1—C9—C8	140.3 (3)
P2—Ru1—P3—C48	−22.42 (17)	C4—Co1—C9—C8	95.8 (4)
Cl1—Ru1—P3—C48	49.36 (15)	C8—C9—C10—C6	0.5 (5)
P1—Ru1—P3—C48	144.29 (15)	Co1—C9—C10—C6	−59.7 (3)
C9—Co1—C1—C5	−60.9 (4)	C8—C9—C10—P2	−178.7 (3)
C10—Co1—C1—C5	−99.7 (3)	Co1—C9—C10—P2	121.2 (3)
C2—Co1—C1—C5	119.2 (5)	C8—C9—C10—Co1	60.2 (3)
C7—Co1—C1—C5	177.4 (3)	C7—C6—C10—C9	0.4 (5)
C3—Co1—C1—C5	82.2 (3)	Co1—C6—C10—C9	59.3 (3)
C6—Co1—C1—C5	−144.7 (3)	C7—C6—C10—P2	179.4 (4)
C4—Co1—C1—C5	38.4 (3)	Co1—C6—C10—P2	−121.6 (4)
C9—Co1—C1—C2	179.9 (4)	C7—C6—C10—Co1	−58.9 (3)
C10—Co1—C1—C2	141.1 (4)	C29—P2—C10—C9	73.0 (4)
C7—Co1—C1—C2	58.2 (5)	C23—P2—C10—C9	178.5 (4)
C3—Co1—C1—C2	−37.0 (4)	Ru1—P2—C10—C9	−50.8 (4)
C6—Co1—C1—C2	96.2 (4)	C29—P2—C10—C6	−105.9 (4)
C5—Co1—C1—C2	−119.2 (5)	C23—P2—C10—C6	−0.4 (5)
C4—Co1—C1—C2	−80.7 (4)	Ru1—P2—C10—C6	130.3 (4)
C5—C1—C2—C3	0.3 (6)	C29—P2—C10—Co1	160.2 (3)
Co1—C1—C2—C3	59.6 (4)	C23—P2—C10—Co1	−94.3 (3)
C5—C1—C2—Co1	−59.3 (3)	Ru1—P2—C10—Co1	36.3 (3)
C10—Co1—C2—C3	177.1 (3)	C2—Co1—C10—C9	−179.5 (3)
C7—Co1—C2—C3	95.6 (4)	C7—Co1—C10—C9	−81.5 (3)
C1—Co1—C2—C3	−119.6 (5)	C1—Co1—C10—C9	140.9 (3)
C6—Co1—C2—C3	138.7 (3)	C6—Co1—C10—C9	−118.4 (4)
C8—Co1—C2—C3	58.6 (5)	C8—Co1—C10—C9	−38.3 (3)
C5—Co1—C2—C3	−81.8 (3)	C5—Co1—C10—C9	96.4 (3)
C4—Co1—C2—C3	−37.3 (3)	C4—Co1—C10—C9	57.4 (4)
C10—Co1—C2—C1	−63.3 (5)	C9—Co1—C10—C6	118.4 (4)
C7—Co1—C2—C1	−144.8 (4)	C2—Co1—C10—C6	−61.1 (4)
C3—Co1—C2—C1	119.6 (5)	C7—Co1—C10—C6	36.9 (3)
C6—Co1—C2—C1	−101.6 (4)	C1—Co1—C10—C6	−100.7 (3)
C8—Co1—C2—C1	178.2 (4)	C8—Co1—C10—C6	80.1 (3)
C5—Co1—C2—C1	37.8 (3)	C5—Co1—C10—C6	−145.2 (3)
C4—Co1—C2—C1	82.3 (4)	C4—Co1—C10—C6	175.9 (3)
C1—C2—C3—C4	−0.1 (6)	C9—Co1—C10—P2	−115.8 (4)
Co1—C2—C3—C4	59.1 (4)	C2—Co1—C10—P2	64.7 (4)
C1—C2—C3—Co1	−59.2 (4)	C7—Co1—C10—P2	162.7 (4)
C9—Co1—C3—C2	−179.5 (3)	C1—Co1—C10—P2	25.1 (4)
C7—Co1—C3—C2	−100.0 (4)	C6—Co1—C10—P2	125.8 (5)
C1—Co1—C3—C2	38.1 (3)	C8—Co1—C10—P2	−154.1 (4)
C6—Co1—C3—C2	−63.8 (5)	C5—Co1—C10—P2	−19.4 (4)
C8—Co1—C3—C2	−142.8 (4)	C4—Co1—C10—P2	−58.4 (5)
C5—Co1—C3—C2	82.1 (3)	C5—P1—C11—C16	102.5 (4)
C4—Co1—C3—C2	120.1 (5)	C17—P1—C11—C16	−154.2 (3)
C9—Co1—C3—C4	60.4 (5)	Ru1—P1—C11—C16	−27.0 (4)
C2—Co1—C3—C4	−120.1 (5)	C5—P1—C11—C12	−77.0 (4)
C7—Co1—C3—C4	139.9 (4)	C17—P1—C11—C12	26.2 (4)
C1—Co1—C3—C4	−82.0 (3)	Ru1—P1—C11—C12	153.5 (3)
C6—Co1—C3—C4	176.1 (3)	C16—C11—C12—C13	2.4 (7)
C8—Co1—C3—C4	97.2 (4)	P1—C11—C12—C13	−178.0 (4)
C5—Co1—C3—C4	−38.0 (3)	C11—C12—C13—C14	−1.9 (9)
C2—C3—C4—C5	−0.2 (6)	C12—C13—C14—C15	0.0 (10)
Co1—C3—C4—C5	58.8 (3)	C13—C14—C15—C16	1.3 (10)
C2—C3—C4—Co1	−59.0 (4)	C14—C15—C16—C11	−0.7 (9)
C9—Co1—C4—C3	−142.7 (4)	C12—C11—C16—C15	−1.1 (7)
C10—Co1—C4—C3	−179.1 (4)	P1—C11—C16—C15	179.3 (4)
C2—Co1—C4—C3	36.9 (4)	C5—P1—C17—C22	−39.0 (4)
C7—Co1—C4—C3	−61.5 (5)	C11—P1—C17—C22	−139.1 (4)
C1—Co1—C4—C3	81.4 (4)	Ru1—P1—C17—C22	84.1 (4)
C8—Co1—C4—C3	−98.4 (4)	C5—P1—C17—C18	147.3 (3)
C5—Co1—C4—C3	119.5 (5)	C11—P1—C17—C18	47.2 (3)
C9—Co1—C4—C5	97.7 (3)	Ru1—P1—C17—C18	−89.6 (3)
C10—Co1—C4—C5	61.4 (4)	C22—C17—C18—C19	1.3 (6)
C2—Co1—C4—C5	−82.7 (3)	P1—C17—C18—C19	175.2 (3)
C7—Co1—C4—C5	178.9 (3)	C17—C18—C19—C20	−1.3 (7)
C1—Co1—C4—C5	−38.1 (3)	C18—C19—C20—C21	−0.1 (7)
C3—Co1—C4—C5	−119.5 (5)	C19—C20—C21—C22	1.5 (7)
C8—Co1—C4—C5	142.0 (3)	C20—C21—C22—C17	−1.5 (7)
C2—C1—C5—C4	−0.4 (5)	C18—C17—C22—C21	0.1 (6)
Co1—C1—C5—C4	−59.3 (3)	P1—C17—C22—C21	−173.6 (4)
C2—C1—C5—P1	179.0 (3)	C29—P2—C23—C24	165.1 (4)
Co1—C1—C5—P1	120.0 (3)	C10—P2—C23—C24	62.2 (4)
C2—C1—C5—Co1	58.9 (3)	Ru1—P2—C23—C24	−69.5 (4)
C3—C4—C5—C1	0.3 (5)	C29—P2—C23—C28	−19.0 (4)
Co1—C4—C5—C1	59.2 (3)	C10—P2—C23—C28	−121.9 (4)
C3—C4—C5—P1	−179.0 (4)	Ru1—P2—C23—C28	106.3 (4)
Co1—C4—C5—P1	−120.1 (3)	C28—C23—C24—C25	2.0 (8)
C3—C4—C5—Co1	−58.9 (3)	P2—C23—C24—C25	178.0 (4)
C11—P1—C5—C1	172.3 (4)	C23—C24—C25—C26	−1.5 (9)
C17—P1—C5—C1	68.6 (4)	C24—C25—C26—C27	−0.2 (9)
Ru1—P1—C5—C1	−51.2 (4)	C25—C26—C27—C28	1.2 (9)
C11—P1—C5—C4	−8.5 (4)	C24—C23—C28—C27	−1.0 (8)
C17—P1—C5—C4	−112.2 (4)	P2—C23—C28—C27	−177.0 (4)
Ru1—P1—C5—C4	128.0 (3)	C26—C27—C28—C23	−0.6 (9)
C11—P1—C5—Co1	−98.0 (3)	C10—P2—C29—C30	16.6 (4)
C17—P1—C5—Co1	158.3 (3)	C23—P2—C29—C30	−89.6 (4)
Ru1—P1—C5—Co1	38.5 (3)	Ru1—P2—C29—C30	143.4 (3)
C9—Co1—C5—C1	142.1 (3)	C10—P2—C29—C34	−165.8 (3)
C10—Co1—C5—C1	98.0 (3)	C23—P2—C29—C34	88.0 (4)
C2—Co1—C5—C1	−38.1 (4)	Ru1—P2—C29—C34	−38.9 (4)
C3—Co1—C5—C1	−81.2 (4)	C34—C29—C30—C31	2.8 (6)
C6—Co1—C5—C1	60.1 (4)	P2—C29—C30—C31	−179.5 (3)
C8—Co1—C5—C1	−178.8 (4)	C29—C30—C31—C32	−3.2 (7)
C4—Co1—C5—C1	−118.6 (4)	C30—C31—C32—C33	1.2 (8)
C9—Co1—C5—C4	−99.3 (3)	C31—C32—C33—C34	1.2 (8)
C10—Co1—C5—C4	−143.4 (3)	C32—C33—C34—C29	−1.6 (7)
C2—Co1—C5—C4	80.5 (4)	C30—C29—C34—C33	−0.4 (7)
C1—Co1—C5—C4	118.6 (4)	P2—C29—C34—C33	−178.2 (4)
C3—Co1—C5—C4	37.4 (4)	C42—P3—C36—C37	164.1 (3)
C6—Co1—C5—C4	178.6 (3)	C48—P3—C36—C37	−87.9 (3)
C8—Co1—C5—C4	−60.2 (4)	Ru1—P3—C36—C37	34.5 (4)
C9—Co1—C5—P1	21.1 (4)	C42—P3—C36—C41	−17.1 (4)
C10—Co1—C5—P1	−23.0 (4)	C48—P3—C36—C41	90.9 (4)
C2—Co1—C5—P1	−159.1 (4)	Ru1—P3—C36—C41	−146.7 (3)
C1—Co1—C5—P1	−121.0 (5)	C41—C36—C37—C38	0.9 (6)
C3—Co1—C5—P1	157.8 (4)	P3—C36—C37—C38	179.8 (3)
C6—Co1—C5—P1	−61.0 (5)	C36—C37—C38—C39	0.3 (7)
C8—Co1—C5—P1	60.2 (4)	C37—C38—C39—C40	−1.4 (7)
C4—Co1—C5—P1	120.4 (5)	C38—C39—C40—C41	1.2 (7)
C9—Co1—C6—C7	81.6 (4)	C37—C36—C41—C40	−1.1 (6)
C10—Co1—C6—C7	119.7 (5)	P3—C36—C41—C40	−179.9 (3)
C2—Co1—C6—C7	−98.0 (4)	C39—C40—C41—C36	0.1 (7)
C1—Co1—C6—C7	−142.8 (3)	C36—P3—C42—C43	−74.3 (3)
C3—Co1—C6—C7	−59.7 (5)	C48—P3—C42—C43	179.9 (3)
C8—Co1—C6—C7	37.0 (3)	Ru1—P3—C42—C43	57.9 (4)
C5—Co1—C6—C7	178.9 (4)	C36—P3—C42—C47	99.3 (4)
C9—Co1—C6—C10	−38.1 (3)	C48—P3—C42—C47	−6.5 (5)
C2—Co1—C6—C10	142.3 (3)	Ru1—P3—C42—C47	−128.5 (4)
C7—Co1—C6—C10	−119.7 (5)	C47—C42—C43—C44	−2.3 (7)
C1—Co1—C6—C10	97.5 (3)	P3—C42—C43—C44	171.7 (4)
C3—Co1—C6—C10	−179.3 (4)	C42—C43—C44—C45	1.7 (7)
C8—Co1—C6—C10	−82.7 (3)	C43—C44—C45—C46	−0.4 (9)
C5—Co1—C6—C10	59.3 (4)	C44—C45—C46—C47	−0.1 (11)
C10—C6—C7—C8	−1.1 (6)	C45—C46—C47—C42	−0.6 (10)
Co1—C6—C7—C8	−59.6 (4)	C43—C42—C47—C46	1.7 (8)
C10—C6—C7—Co1	58.5 (3)	P3—C42—C47—C46	−171.9 (5)
C9—Co1—C7—C6	−81.9 (3)	C42—P3—C48—C53	−63.1 (4)
C10—Co1—C7—C6	−38.0 (3)	C36—P3—C48—C53	−167.6 (4)
C2—Co1—C7—C6	98.2 (4)	Ru1—P3—C48—C53	63.0 (4)
C1—Co1—C7—C6	61.2 (5)	C42—P3—C48—C49	118.4 (4)
C3—Co1—C7—C6	141.2 (4)	C36—P3—C48—C49	13.9 (4)
C8—Co1—C7—C6	−120.1 (5)	Ru1—P3—C48—C49	−115.5 (4)
C4—Co1—C7—C6	178.6 (3)	C53—C48—C49—C50	−0.1 (7)
C9—Co1—C7—C8	38.2 (3)	P3—C48—C49—C50	178.4 (4)
C10—Co1—C7—C8	82.1 (3)	C48—C49—C50—C51	1.1 (8)
C2—Co1—C7—C8	−141.6 (4)	C49—C50—C51—C52	−1.1 (9)
C1—Co1—C7—C8	−178.6 (4)	C50—C51—C52—C53	0.2 (10)
C3—Co1—C7—C8	−98.7 (4)	C49—C48—C53—C52	−0.8 (8)
C6—Co1—C7—C8	120.2 (5)	P3—C48—C53—C52	−179.3 (5)
C4—Co1—C7—C8	−61.2 (5)	C51—C52—C53—C48	0.7 (10)

### Hydrogen-bond geometry (Å, °)

**Table:**

*D*—H···*A*	*D*—H	H···*A*	*D*···*A*	*D*—H···*A*
C3—H3···F2^i^	0.93	2.35	3.230 (9)	157
C7—H7···F3^i^	0.93	2.51	3.383 (8)	156
C16—H16···Cl1	0.93	2.57	3.414 (5)	151
C37—H37···Cl1	0.93	2.77	3.584 (4)	147
C46—H46···F2^ii^	0.93	2.54	3.219 (10)	130
C55—H55A···F3^iii^	0.97	2.42	3.140 (12)	131

Symmetry codes: (i) −*x*+1, −*y*, *z*−1/2; (ii) *x*+1/2, −*y*+1/2, *z*; (iii) −*x*+1, −*y*, *z*+1/2.

## References

[bb1] Bruker (2007). *SMART* and *SAINT* Bruker AXS Inc., Madison, Wisconsin, USA.

[bb2] Flack, H. D. (1983). *Acta Cryst.* A**39**, 876–881.

[bb3] Santos, A., Montoya, J., Noheda, P., Romero, A. & Echavarren, A. M. (1994). *Organometallics*, **13**, 3605–3615.

[bb4] Sheldrick, G. M. (1996). *SADABS* University of Göttingen, Germany.

[bb5] Sheldrick, G. M. (2008). *Acta Cryst.* A**64**, 112–122. 10.1107/S0108767307043930 18156677

